# Acquired Type 1 Brugada Syndrome Induced by Chronic High-Dose Kratom Use

**DOI:** 10.7759/cureus.72129

**Published:** 2024-10-22

**Authors:** Kenneth Holton, Christian Sanchez Corredera, Jason Haidar, Ramon G Valentin, Donny Perez, Eric Boccio

**Affiliations:** 1 Department of Emergency Medicine, Memorial Healthcare System, Hollywood, USA; 2 Department of Emergency Medicine, Nova Southeastern University Dr. Kiran C. Patel College of Osteopathic Medicine, Fort Lauderdale, USA; 3 Department of Emergency Medicine, Florida International University Herbert Wertheim College of Medicine, Miami, USA; 4 Department of Critical Care Medicine, Memorial Healthcare System, Hollywood, USA

**Keywords:** brugada syndrome, case report, kratom cardiotoxicity, kratom toxicity, mitragyna speciosa

## Abstract

Kratom denotes the naturally occurring alkaloids found in *Mitragyna speciosa* and is self-administered for the treatment of chronic pain, anxiety, and opioid withdrawal. Kratom is cardiotoxic with several case reports linking its use to QTc prolongation and ventricular dysrhythmias; however, there exist few reports of kratom-induced Brugada syndrome.

A 36-year-old male with a past medical history inclusive of polysubstance abuse, bipolar disorder, and seizure disorder presented to the emergency department for chest pain. The patient reported a three-week history of lightheadedness when standing, oftentimes accompanied by palpitations and syncope. The patient was prescribed lamotrigine but reported non-adherence for more than three months due to a lack of insurance. The patient self-administered high-dose (~100 grams/day) white vein kratom daily for the last three years for symptoms of opioid withdrawal and alcohol cravings. He denied a family history of sudden cardiac death. The initial electrocardiogram (EKG) demonstrated coved ST segments followed by a negative T wave in leads V1 and V2 consistent with a type 1 Brugada pattern. Pacer pads were applied, and cardiology and electrophysiology were consulted. The patient was admitted to the medical intensive care unit on telemetry. Serial EKGs returned to normal sinus rhythm, and an inpatient flecainide challenge was negative, making Brugada syndrome secondary to chronic high-dose kratom use the most likely diagnosis.

We presented a case of type 1 Brugada syndrome induced by chronic high-dose kratom use. While the clinical management of kratom toxicity is mostly supportive, physicians should consider potentially fatal cardiac dysrhythmias including Brugada syndrome.

## Introduction

Kratom is an herbal extract derived from the leaves of the evergreen tree *Mitragyna speciosa* [1]. Kratom is a supplement available over the counter and is self-administered for chronic pain, anxiety, and fatigue. It is commonly used to manage drug withdrawal symptoms and cravings but is becoming increasingly popular as a recreational drug [2]. Kratom toxicity presents clinically as agitation, anxiety, tachycardia, and palpitations but has also been implicated in new-onset seizures and cardiac dysrhythmias [3]. While the most common cardiotoxic effect of kratom is QTc prolongation, a small number of case reports highlighting kratom-induced Brugada syndrome have been described. We present a case of acquired type 1 Brugada syndrome induced by chronic high-dose kratom use.

## Case presentation

A 36-year-old male with a past medical history including alcohol abuse (last drink three months ago), tobacco use (three packs/day) with an 18-year pack history, opioid use disorder, bipolar disorder, depression, and seizure disorder presented to the emergency department for chest pain. The chest pain was described as sharp, intermittent, radiating to the left shoulder, present at rest, and exacerbated by exertion. Multiple episodes occurred daily over the last four weeks with increasing duration and frequency. The patient reported associated lightheadedness when transitioning from sitting to standing, oftentimes accompanied by palpitations, left upper extremity paresthesia, and loss of consciousness, which prompted him to seek medical attention. Syncopal episodes lasted several seconds and were always preceded by palpitations. The patient was prescribed lamotrigine, but he reported non-adherence for more than three months due to a lack of insurance. The patient admitted to self-administering high-dose (~100 grams/day) white vein kratom four times daily in the form of swallowed powder over the last three years to manage symptoms of polysubstance withdrawal and cravings, having previously declined Suboxone. He denied personal and family histories of cardiac dysrhythmia and sudden cardiac death.

Initial vital signs revealed hypertension (115/114 millimeters of mercury) and tachycardia (106 beats/minute). The patient's respiratory rate, peripheral capillary oxygen saturation, and temperature were within normal limits (18 breaths/minute, 98% on room air, and 37.4 degrees Celsius oral, respectively). On arrival, the patient was awake, alert, and oriented to self, place, and time with a corresponding Glasgow Coma Scale score of 15. The patient was in no acute distress and appeared non-toxic. Physical examination was remarkable for left-sided chest pain that radiated to the left clavicle and neck that was not reproducible with palpation. The rate and rhythm were fast and regular, respectively. There were no rubs, gallops, or murmurs appreciated on cardiac auscultation. Lung sounds were clear to auscultation bilaterally. The abdomen was soft, non-tender, and non-distended. The remainder of the physical examination was unremarkable.

**Figure 1:**
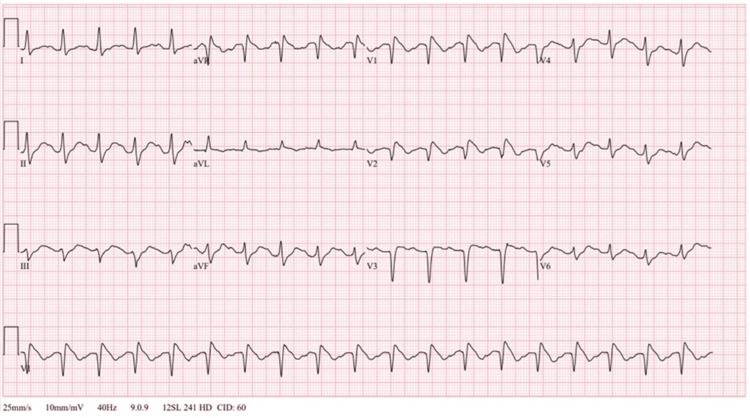
Initial emergency department 12-lead electrocardiogram showing coved ST segment elevations >2 millimeters in leads V1 and V2 consistent with type 1 Brugada pattern

The initial electrocardiogram (EKG) demonstrated tachycardia (114 beats/minute) with a normal QTc (490 milliseconds) and coved ST segments followed by T wave inversions in leads V1 and V2 consistent with type 1 Brugada pattern (Figure 1).

**Figure 2:**
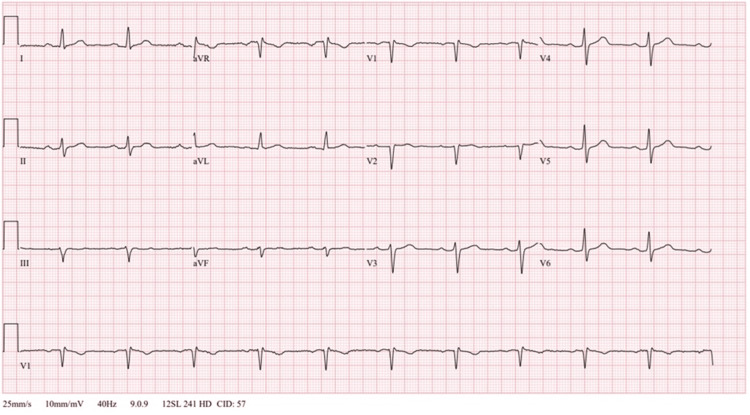
Repeat electrocardiogram performed on hospital day 3 showing resolution of Brugada pattern and return to normal sinus rhythm

A complete blood count was remarkable for leukocytosis (14,500 cells/microliter (µL)) (reference range: 3,500-10,000/µL). A comprehensive metabolic panel revealed normal sodium (141 millimoles/liter (mmol/L)) (reference range: 137-145 mmol/L), potassium (3.5 mmol/L) (reference range: 3.5-5.1 mmol/L), chloride (106 mmol/L) (reference range: 98-107 mmol/L), and calcium (8.4 milligrams/deciliter (mg/dL)) (reference range: 8.4-10.2 mg/dL) levels. Magnesium and phosphorus levels were within normal limits (1.7 mg/dL (reference range: 1.6-2.3 mg/dL) and 3.8 mg/dL (reference range: 2.5-4.5 mg/dL), respectively). Initial and serial troponins were within normal limits (<0.012 nanograms/milliliter (ng/mL)). Thyroid-stimulating hormone and free T4 levels were within normal limits (1.100 international units (IU)/mL (reference range: 0.465-4.680 IU/mL) and 1.08 ng/dL (reference range: 0.89-1.76 ng/dL), respectively). Pacer pads were applied to the patient, and interventional cardiology and electrophysiology were consulted. The patient was admitted to the medical intensive care unit on telemetry. A formal cardiac echocardiography demonstrated normal function without structural pathology. On hospital day 3, telemetry and serial EKGs returned to normal sinus rhythm (Figure 2).

An inpatient flecainide challenge was negative, and automated implantable cardioverter defibrillator (AICD) placement was not indicated. The patient was counseled regarding kratom use and offered resources for opioid use disorder. The patient declined Suboxone, and an outpatient referral to pain management was arranged.

## Discussion

*Mitragyna speciosa* is a tropical plant belonging to the Rubiaceae family and is native to Southeast Asia. Colloquially known as kratom, the plant species has traditionally been used for medicinal purposes in treating conditions such as fever, diarrhea, diabetes, pain, and fatigue and as a wound poultice. Kratom use has become increasingly popular as a cheap alternative to opioids and is commonly taken as a self-treatment remedy for opioid withdrawal symptoms and cravings [2]. Several strains of kratom have been identified and include red, green, and white based on the color of the leaf vein. Reports have shown varying effects and preferences for different kratom strains among users [4]. Kratom can be consumed in several forms; fresh leaves can be chewed, ground into powders, swallowed, dried for smoking, or steeped as tea. Commercially available products can be purchased over the counter or ordered online and commonly exist in powder form.

*Mitragyna speciosa* is known to contain over 40 different alkaloids, four of which are known to be pharmacologically active: mitragynine, 7-hydroxymitragynine (7-OH-mitragynine), speciociliatine, and corynantheidine [1]. The mechanism of interaction of kratom alkaloids at opioid receptors is disputed, and several models have been posited. While several suggest mitragynine and 7-OH-mitragynine behave as agonists with µ- and δ-receptors and µ- and κ-receptors, respectively, competing arguments suggest mitragynine and 7-OH-mitragynine are partial agonists at µ-receptors and competitive antagonists at δ-receptors and have negligible effects on κ-receptors [5]. The metabolism of kratom alkaloids occurs primarily in the liver, suggesting possible implications for CYP450 drug interactions [6]. Mitragynine has been shown to prolong the action potential in human-induced pluripotent stem cell-derived cardiomyocytes through its interaction with the rapidly delayed rectifier potassium current [7]. This channel is involved with cardiac repolarization, and its inhibition has been postulated to increase the likelihood of torsade de pointes.

Kratom toxicity presents clinically as nausea, vomiting, tremors, diaphoresis, tachycardia, palpitations, hypertension, and elevated creatinine phosphokinase levels. The treatment for kratom toxicity is mainly supportive and includes intravenous fluids, sedatives, antiemetics, and, in rare cases, antihypertensives and supplemental oxygen. Regular kratom use may lead to psychological and physical dependence, which may precipitate withdrawal symptoms. Psychological withdrawal symptoms include depressed mood, anxiety, restlessness, and irritability, while physical symptoms are similar to that of opioid withdrawal and include lacrimation, rhinorrhea, piloerection, myalgias, joint pain, diarrhea, nausea, vomiting, insomnia, autonomic hyperactivity, and yawning [3]. Therapeutic management is mainly supportive and may include administration of buprenorphine, naloxone, and clonidine.

While the most common cardiotoxic effect of kratom is QTc prolongation leading to torsade de pointes, a small number of case reports describing Brugada syndrome in the setting of chronic or high-dose kratom use can be found. Brugada syndrome is a familial autosomal dominant channelopathy characterized by an alteration of transmembrane ion currents of cardiac cells. Patients with Brugada syndrome are prone to ventricular arrhythmias and may experience sudden cardiac arrest during periods of exertion and while asleep. Brugada syndrome has been linked to mutations in genes coding sodium and calcium transporters, notably *SCN5A*, *CACNA1C*, and *CACN2b* [8,9]. Cases of acquired Brugada syndrome in the setting of antiarrhythmic sodium and calcium channel blockers are well described and represent the basis for drug provocation tests used to assess individual risk for and prognosis of Brugada syndrome. Brugada syndrome is a diagnosis that requires both the presence of clinical symptoms and characteristic EKG findings. Symptoms include syncope, lightheadedness, chest pain, shortness of breath, palpitations, seizures, and sudden death. There are three types of Brugada pattern on EKG; type 1 consists of coved ST segment and J point elevation of at least 2 millimeters in at least two of the precordial EKG leads (V1-V3), type 2 has saddleback shaped ST elevation of at least 2 millimeters, and type 3 can share the morphology of either type 1 or 2 but with less than 2 millimeters of ST segment elevation. The diagnosis of Brugada syndrome is established by either observing a spontaneous type 1 EKG pattern or unmasking a type 1 pattern with a drug challenge typically induced with a sodium channel blocker in suspected individuals [10]. Current guidelines recommend AICD placement only in patients with spontaneous type 1 EKG pattern and either a history of aborted cardiac arrest or documented sustained ventricular arrhythmias or syncope of arrhythmic origin [11].

A recent literature search yielded only three reported cases of Brugada syndrome in the setting of frequent high-dose kratom use [12-14]. A comprehensive review of the adverse cardiovascular effects and cardiotoxicity of kratom published in 2021 discussed the increased risk of QTc prolongation, torsades de pointes, and cardiopulmonary arrest but failed to mention any association with Brugada syndrome [15]. The Brugada syndrome cases unmasked by kratom had remarkable similarities. All individuals had been taking kratom for either an extended duration (>12 months) or consuming the powder form in high doses. One patient presented with new-onset dizziness, the second with syncope, and the third with new-onset seizure. None of the individuals reported a personal or family history of Brugada syndrome or sudden cardiac death. All patients met the diagnostic criteria of Brugada syndrome given their presenting symptoms and EKGs demonstrating a type 1 Brugada pattern. Laboratory testing and diagnostic imaging including echocardiography were reported normal for all cases. One subject underwent electrophysiological testing, which failed to induce any ventricular arrhythmias. Repeat EKGs performed 24-48 hours after the initial presentation had all returned to normal sinus rhythm. Patients were educated regarding kratom, asked to refrain from its use, and discharged without sequelae. Given the frequency and similarities between the case reports, the link between frequent, chronic, and high-dose kratom use and Brugada syndrome is convincing.

## Conclusions

We presented a case of acquired type 1 Brugada syndrome induced by chronic high-dose kratom use. Kratom's cardiotoxic effects relating to QTc prolongation and torsades de pointes have been well described; however, the mechanism of its interaction with sodium channels and influence on the risk of inducing type 1 Brugada syndrome are less understood and warrant further research. Given the growing popularity, ease of access, and lack of regulation and oversight surrounding kratom use, healthcare professionals must be aware of its potential risks, especially in regard to potentially fatal cardiac dysrhythmias.
